# White matter disconnection impacts proprioception post-stroke

**DOI:** 10.1371/journal.pone.0310312

**Published:** 2024-09-12

**Authors:** Matthew Chilvers, Trevor Low, Deepthi Rajashekar, Sean Dukelow

**Affiliations:** 1 Department of Clinical Neurosciences, Cumming School of Medicine, University of Calgary, Calgary, Alberta, Canada; 2 Hotchkiss Brain Institute, University of Calgary, Calgary, Alberta, Canada; Universita degli Studi di Torino, ITALY

## Abstract

Proprioceptive impairments occur in approximately 50–64% of people following stroke. While much is known about the grey matter structures underlying proprioception, our understanding of the white matter correlates of proprioceptive impairments is less well developed. It is recognised that behavioural impairments post-stroke are often the result of disconnection between wide-scale brain networks, however the disconnectome associated with proprioception post-stroke is unknown. In the current study, white matter disconnection was assessed in relation to performance on a robotic arm position matching (APM) task. Neuroimaging and robotic assessments of proprioception were collected for 203 stroke survivors, approximately 2-weeks post-stroke. The robotic assessment was performed in a KINARM Exoskeleton robotic device and consisted of a nine-target APM task. First, the relationship between white matter tract lesion load and performance on the APM task was assessed. Next, differences in the disconnectome between participants with and without impairments on the APM task were examined. Greater lesion load to the superior longitudinal fasciculus (SLF II and III), arcuate fasciculus (all segments) and fronto-insular tracts were associated with worse APM task performance. In those with APM task impairments, there was, additionally, disconnection of the posterior corpus callosum, inferior fronto-occipital fasciculus, inferior longitudinal fasciculus and optic radiations. This study highlights an important perisylvian white matter network supporting proprioceptive processing in the human brain. It also identifies white matter tracts, important for relaying proprioceptive information from parietal and frontal brain regions, that are not traditionally considered proprioceptive in nature.

## Introduction

Proprioception, often considered the “sixth sense”, describes the perception of the location and movement of our limbs, arising from within the body itself [1]. Proprioception plays an important role in the accurate control of movement, integrating with vision to allow us to move freely within and interact with our environment [2]. Approximately 50–64% of stroke survivors experience proprioceptive impairments [3–5]. These impairments have been linked with poorer outcomes, including increased hospital stays, reduced independence, reduced participation in activities of daily living and impaired motor learning [5–9]. Despite the clinical importance of proprioception, its underlying neuroanatomy is not fully understood, particularly regarding the central white matter tracts. Considering white matter makes up around 50% of brain volume [10], understanding the white matter correlates of proprioceptive impairment after stroke is important.

Within the central nervous system, our knowledge of proprioceptive neuroanatomy is evolving. Several important investigations have focused on better understanding cortical structures underlying proprioceptive function in neurologically intact participants [11–16] and in stroke participants [17–21]. This work has consistently shown that many cortical regions beyond somatosensory cortex (S1) are important for proprioceptive tasks [17–24].

Perhaps the least studied aspect of the nervous system involved in proprioception is the central white matter. While the role of the ascending Dorsal Column Medial Lemniscus (DCML) and dorsal spinocerebellar pathways are well documented, less is known about the white matter connections between cortical areas involved in proprioceptive processing. Diffusion imaging techniques have started to investigate the importance of cortico-cortical white matter tracts for proprioception post-stroke [25, 26]. Other studies have suggested the involvement of other white matter tracts in proprioception, such as the superior longitudinal fasciculus (SLF), by studying the functional activity of the cortical regions connected by white matter structures using fMRI [17, 24, 27].

Together, studies on the grey and white matter of proprioception are demonstrating that proprioceptive processing is, perhaps, more complex than initially thought, especially regarding cortico-cortical connections. To better understand these complexities, it is crucial to further our understanding of the cortico-cortical white matter connections that support proprioception in the human brain.

The understanding that distant regions communicate through long and short white matter connections, to produce intricate functions, has led to the study of their disconnection. This line of study links disruptions to interconnected brain networks with given symptoms and syndromes [28–31]. While disconnection research was originally restricted to post-mortem investigation, modern neuroimaging techniques have allowed single white matter tracts to be imaged in the living human brain [32–35]. This has enabled investigations into the functional correlates of human white matter disconnection following stroke, and has been used in the study of motor deficits [36, 37], somatosensory deficits [38], attentional deficits [39–41], cognitive function [37, 42], language [43–45], category fluency deficits [46], anosognosia for hemiplegia [47] and fatigue [48] post-stroke. The relationship between white matter disconnection and proprioceptive impairments post-stroke remains relatively unknown.

The aim of the current study was to assess the impact of white matter disconnection, following stroke, on performance of a robotic Arm Position Matching (APM) task.

## Materials and methods

### Participant recruitment

**Fig 1:**
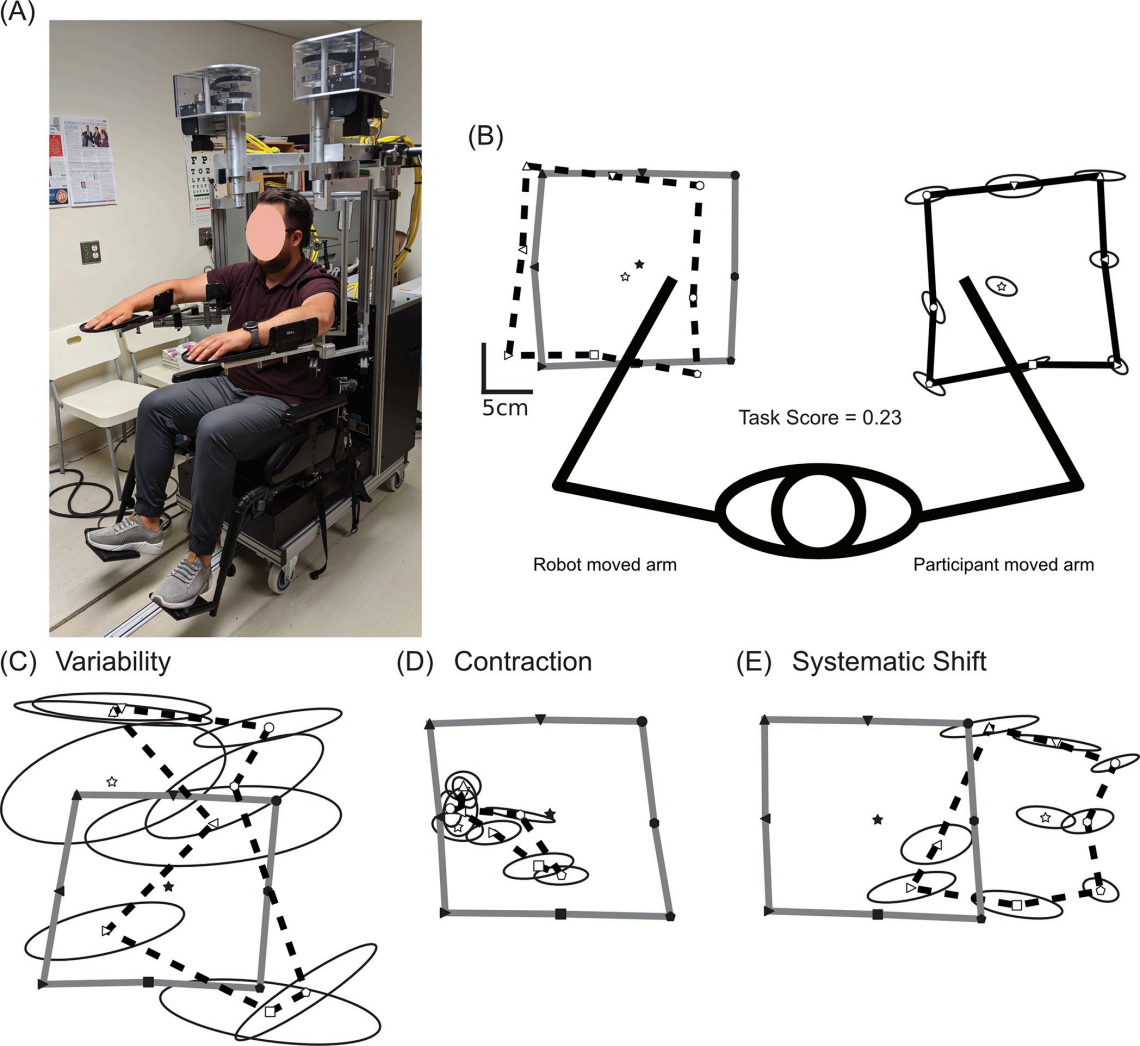
Methods. (A) The Kinarm Exoskeleton robotic device used to perform the Arm Position Matching (APM) Task. (B) An exemplar of the APM Task. Data taken from a stroke participant with normal performance (Task Score of 0.23). In this instance, the robot moved the participants stroke affected, left arm. Each filled symbol represents the mean position of one the nine targets where the robot moved the participant’s affected arm. The participant mirror-matched with the unaffected, right arm. The open symbols represent the mean position of each target where the participant moved their unaffected arm. Ellipsoids represent one standard deviation of variability around each matched position. For illustrative purposes the grey and solid black outlines connect the mean hand positions of the outer 8 targets for the robotically moved affected arm and participant moved arm, respectively. The data from the participant moved arm is also reflected across the midline of the participant, to make easier comparison to the target locations of the robotically moved passive arm (open symbols, outer targets connected by black dashed line). (C-E) Similar to (B), these panels demonstrate the participant’s attempts to match with the active arm (open symbols) reflected across the midline for comparison to the robotically moved hand positions (closed symbols). This has been done for illustrative purposes so the reader can make a direct visual comparison. Exemplars from stroke participants illustrate high amounts of variability (C), contraction (D) and systematic shift (E). *It should be noted that while panel D) displays a participant with high amounts of contraction*, *it is also possible to observe examples of expansion of the workspace*.

Participants were recruited from the inpatient stroke units at either the Foothills Medical Centre, or Carewest Dr. Vernon Fanning Centre, both in Calgary, Alberta, Canada. Participants were included in the current study if they were: 18 years of age or older, had a first time ischaemic or haemorrhagic unilateral stroke and were able to follow the instructions to complete the robotic and clinical testing. Participants were excluded from the current study if they had: lesions impacting the cerebellum or below the level of the midbrain (due to the likelihood of ipsilesional sensory impairments caused by lesions to these structures), other neurological disorders (such as multiple sclerosis, Parkinson’s disease etc.), apraxia [49], or significant upper extremity orthopaedic injury and/or pain. Since participants had to understand the task instructions, participants with receptive aphasia, who potentially may have had larger left hemisphere lesions, were also excluded. All participants provided written informed consent, in accordance with the Declaration of Helsinki. The study was approved by the Conjoint Health Research Ethics Board at the University of Calgary (REB15-1340). The individual in Fig 1 in this manuscript has given written informed consent (as outlined in the PLOS consent form) to publish these case details.

### Neuroimaging acquisition

Clinical imaging was collected for all participants in accordance with the acute stroke protocols at the Foothills Medical Centre and included MRI or CT imaging. For MRI, acquisition sequences included: T2-weighted fluid attenuated inversion recovery (FLAIR), diffusion weighted imaging (DWI) and, where appropriate, gradient echo (GRE) or susceptibility weighted imaging (SWI). MRI images were acquired using either a 1.5T or 3T General Electric Medical Systems scanner. CT images were acquired using a Siemens system, or one of three General Electric scanners. The time from stroke to image acquisition was 2.2 ± 3.0 days.

### Lesion delineation and registration

Lesions were identified and marked by trained assessors on the original FLAIR or CT images, using the MRIcron software [50]. Where possible, lesion markings were guided by the DWI. The accuracy of the lesion markings were verified by a neurologist, before being normalised to MNI space. Normalisation was conducted using the clinical toolbox [51] (https://www.nitrc.org/projects/clinicaltbx) in SPM 12 (https://www.fil.ion.ucl.ac.uk/spm/software/spm12/). The resulting normalised lesions were visually inspected for accuracy. Additionally, the lesion markings were used to compute the grey matter lesion volume for each participant, by calculating the extent of overlap between the lesion marking and grey matter segmentation. This segmentation was performed using the FAST tool in FSL [52]. Lesion overlap maps and multi-slice images of our disconnectome analysis result (see below) were created using the free software, MRIcroGL (https://www.nitrc.org/projects/mricrogl).

### BCB toolkit

To quantify the white matter disconnection in stroke participants, the BCB Toolkit [46] (http://www.toolkit.bcblab.com) was used to generate two different metrics of white matter disconnection: white matter tract lesion load and voxel-wise disconnectome maps.

### White matter tract lesion load

The first disconnection metric was a measure of lesion load to different white matter tracts. For each participant, the normalised lesion markings were mapped onto a white matter atlas [53] using the Tractotron tool, as part of the BCBtoolkit [46]. The lesion load to each white matter tract was quantified by measuring the proportion of each white matter tract that was impacted by the lesion. It was calculated by dividing the volume of damaged voxels of each white matter tract in the atlas by the total volume of the tract and expressed as a percentage. While lesion load measures give a simple indication of the extent of damage to white matter tracts, they are not without their limitations [54]. Signal propagation in white matter can be equally interrupted by lesion loads of differing sizes (e.g. a small lesion that perfectly bisects a tract vs a large lesion which destroys the entire tract [55]). Lesion load measures do not account for this, assuming that greater lesion loads to white matter will increase the severity of disconnection [54]. To combat this limitation of lesion load metrics, white matter disconnections were also quantified with an additional voxel-based measure (see section “Disconnectome maps” below).

### Disconnectome maps

Disconnectome maps were used as a second measure of white matter disconnection and describe the disruption in connectivity at the voxel/cluster-based level. This methodology uses tractography, performed in controls, to estimate the white matter streamlines which would, in theory, pass through a given stroke participants lesion. For each stroke participant it quantifies the probability that the white matter, at any given voxel, is disconnected as a result of their lesion. Disconnectome maps were generated using the Disconnectome Maps tool in the BCBtoolkit [46] (http://www.toolkit.bcblab.com), as has previously been used to investigate motor deficits and category fluency in stroke participants [36, 46]. The toolkit relies on a diffusion imaging dataset of 10 healthy control participants, collected elsewhere. *See* [46, 53, 56] *for details*.

Each stroke participant’s lesion was normalised into native space for each of the 10 control participants [57] and subsequently used as a seed region for tractography in each control participant. For every lesion, tractography was performed on each of the control participants diffusion data, using Trackvis [58]. Tractography was conducted as part of the toolbox, as previously described by other authors [59]. This resulted in 10 visitation maps for each stroke participant/lesion, which identified the white matter streamlines that would pass through each stroke participants lesion, based on each of the control participants. These 10 maps were binarised and then summed to create a disconnectome map for each participant. Thus, the disconnectome maps represented the probability of white matter disconnection at every voxel, with values from 0–100% [60]. The subsequent maps were then used for whole-brain analysis, using the Randomise tool in FSL [61]. The advantage of this methodology is that it can quantify disconnection in regions that are distant from, but would otherwise be connected by, white matter passing through the lesion site.

### Robotic assessment of proprioception

All participants completed a robotic Arm Position Matching (APM) Task [62]. This task has been used extensively to quantify limb position sense in adult and paediatric stroke, brain injury, healthy aging, and paediatric populations [5, 63–66]. Robotic assessments were performed using a Kinarm Exoskeleton robotic device (Fig 1A) (Kinarm, Kingston, Ontario, Canada). Robotic assessments were performed 11.2 days ± 7.9 days post-stroke (mean ± standard deviation). Participants sat in the wheelchair base of the robotic device, and their arms were supported against gravity, in the horizontal plane, by arm troughs. The linkages on the robotic device were then adjusted to fit each participant. Once each participant was set up appropriately, they were wheeled into the virtual reality environment. The APM Task was performed in the absence of vision, so once a demonstration of the task had been given and the participant understood the task, vision of the upper limb was occluded by an opaque shutter and a bib placed over the shoulders.

#### Arm position matching task

The APM Task began with the robotic device moving the participants stroke affected arm to one of nine spatial locations, in a pseudorandomized order. Participants were instructed to wait until the robot had finished moving their arm, before attempting to mirror-match the final position of their stroke affected arm with their opposite arm. Once the participant felt that they had their arms in a mirror-matched position, they verbally declared this information to the robot operator. The robot operator then cued the robotic device to move onto the next trial and the robot moved the participants stroke affected arm to the next location. The nine target locations were oriented in a square, with eight outer targets surrounding a ninth central target (Fig 1B). The central target was located in a position at which the participants shoulder and elbow were flexed at 30° and 90° respectively. The APM Task was performed in a block design, with each target location being tested once in each of the six blocks. As such, there were 54 trials in total.

#### APM task score calculation

Performance on the APM Task was quantified by a global measure of performance called the APM Task Score. Robotic analysis was performed in Dexterit-E version 3.9 (Kinarm, Kingston, Ontario, Canada). The first step in calculating APM Task Score was to measure performance on a number of task parameters: *Absolute error* was calculated as the mean absolute distance in mirrored arm position between each arm in the x and y directions. *Variability* (Fig 1C) describes the consistency in ability to match the hand position and was calculated as the mean of the standard deviations in hand positions for each of the nine targets, in both the x and y directions. *Contraction/Expansion* (Fig 1D) describes the perception of the area moved by the robot as being shrunken or enlarged. For the x direction, it was calculated by taking the absolute distance between the mean position of the three left-most and three right-most targets of both the participant and robot moved arms and calculating the ratio between the distance for each arm. The same calculation was conducted for the y direction, except the proximal-most and distal-most targets were used. *Systematic Shift* (Fig 1E) describes the perception that the arm position was more left/rightward or distal/proximal. It was calculated as the mean difference in the x and y positions of the robot and participant moved arms. Full details on these parameters, their calculations and contributions to the APM Task Score are also freely available at: https://kinarm.com/download/kst-summary-analysis-version-3-9/.

Once these parameters were calculated for each participant, they were next compared to a normative model, comprised of 2227 assessments from 799 control participants with no history of neurological disorders. Each parameter score was converted into a standardized score (Z-score), accounting for the effects of age, sex and handedness. Next, the direction of optimal performance for each parameter was determined (i.e. do large positive and/or negative Z-scores indicate best performance). For one-sided parameters (absolute error, variability and systematic shift), Z-scores were transformed to Zeta-scores, such that a score of zero indicated the best possible performance. For two-sided parameters (contraction/expansion), where the best performance was already a score of zero, no zeta-transformation was applied and it was left as a Z-score. The root sum square (RSS) distance was then calculated from all of the parameter Z or Zeta scores. The RSS distance was then transformed into a Z-score using the same methodology as for each parameter score and the subsequent Z-score transformed into a one-sided statistic with zero indicating the best possible APM Task Score. A key feature of the APM Task Score is that it can be used to compare performance to a control participant of the same age, sex and handedness and shares the same percentiles as a normal distribution. As such, APM Task Scores outside of the 95^th^ percentile, or an APM Task Score >1.96 were used to indicate impairments on the APM Task. Further details on all Task Score calculations can be found at: https://kinarm.com/download/kst-summary-analysis-version-3-9/.

### Clinical assessment

In addition to the APM Task, participants completed a battery of clinical assessments to highlight the clinical characteristics of the group. These assessments were conducted by a trained therapist, during the same session as the robotic assessment. The Thumb Localization Test (TLT) was performed to assess for proprioceptive impairments in each participant, with scores ranging from 0–3 [67]. Scores greater than 0 indicated proprioceptive impairment. The Chedoke McMaster Stroke Assessment (CMSA) was performed to assess motor impairment on both the contralesional and ipsilesional arms, with scores less than 7 used to infer motor impairment [68]. The Behavioural Inattention Test (BIT) was performed to assess for hemispatial neglect, with scores less than 130 indicative of neglect [69]. Finally, the Functional Independence Measure (FIM) was conducted to assess participants ability to perform activities of daily living [70]. While these clinical assessments were not used in the subsequent analysis, they were collected to provide a greater insight as to the clinical presentation of the participants in the current study.

### Statistical analysis

The first analysis compared APM Task Scores between participants with left and right hemisphere lesions. Given that APM Task Scores and lesion volume have previously demonstrated a linear relationship [23, 71], a one-way ANCOVA was performed to address potential lesion volume differences. In the APM task, participants matched the limb positions with their ipsilesional arm. Therefore, any ipsilesional impairments could impact participants ability to perform the APM Task. As such, a chi-square test was conducted to assess potential differences in the presence of ipsilesional motor impairments between those with left and right hemisphere lesions.

The second analysis, performed independently for each white matter tract (23 in total), was a multivariate linear regression, to evaluate the relationship between white matter tract lesion load and APM Task Scores. Due to the potential lateralization of proprioception [72, 73] and the known relationships between lesion volume, the extent of damage to grey matter regions and proprioception [18, 20, 23, 71], lesion side and grey matter lesion volume were included as covariates (an additional uncontrolled analysis was also performed). Each variable was first normalised to zero mean and unit standard deviation (SD = 1). To ensure data pertaining to each tract was not sparse, only tracts with more than 25 participants with damage to both the left and right tracts were assessed [74]. Table 1 displays all the tracts included in the atlas, and those with sufficient data points that were included in the analysis (*non-italicised tract names*). To correct for multiple comparisons, the tests on the partial regression coefficients were subject to False Discovery Rate (FDR) (5%) correction (Benjamini-Hochberg procedure) and those which survived the correction were deemed significant. A total of 69 comparisons were accounted for.

**Table 1:** White matter tracts.

Atlas white matter tracts	
Anterior Commissure	*Fronto Insular Tract 2*
Anterior Thalamic Projections	*Fronto Insular Tract 3*
**Arcuate Fasciculus Anterior Segment**	Fronto Insular Tract 4
**Arcuate Fasciculus Long Segment**	**Fronto Insular Tract 5**
Arcuate Fasciculus Posterior Segment	*Fronto Marginal Tract*
Cingulum	Fronto Striatal
*Cingulum Anterior*	Hand Inferior U Tract
*Cingulum Posterior*	*Hand Middle U Tract*
**Corpus Callosum**	*Hand Superior U Tract*
Corticospinal Tract (CST)	**Inferior Fronto-Occipital Fasciculus (IFOF)**
*Face U Tract*	Inferior Longitudinal Fasciculus (ILF)
Fornix	**Optic Radiations**
Frontal Aslant Tract	*Paracentral U Tract*
Frontal Commissural	Pons
*Frontal Inferior Longitudinal*	Superior Longitudinal Fasciculus I (SLF I)
*Frontal Orbito Polar*	**Superior Longitudinal Fasciculus II (SLF II)**
Frontal Superior Longitudinal U Tract	**Superior Longitudinal Fasciculus III (SLF III)**
*Fronto Insular Tract 1*	*Uncinate*

White matter tracts included in the atlas used in the present study [53]. To ensure each regression model had sufficient data pertaining to each side of the brain, only tracts with more than 25 participants with damage to both the left and right tracts were assessed [74]. Italicised tracts did not have sufficient participants with damage in both the left and right tracts (n < 25), thus were excluded from the analysis. Tracts in bold indicates those tracts that were implicated in proprioceptive impairments and appear in Fig 5.

Due to collinearity concerns, an additional principal component regression analysis was performed. First, the predictor variables (white matter tract lesion load and grey matter lesion volume) were normalized and subject to a principal component analysis (PCA), resulting in independent, linear combinations of predictors [75, 76]. PCA has successfully been applied as a dimensionality reduction technique, in neuroimaging studies on many different neuropsychological disorders [75, 77], including stroke [36, 78]. The number of principal components (PC) explaining 95% of variance was then determined and the PC scores from each of these components was entered into a multivariate linear regression. Scores from nine out of a total 24 PCs were entered into the model. Again, tests on the nine partial regression coefficients were subject to a 5% FDR correction.

The final analysis assessed for voxel-wise differences in white matter disconnection between participants with APM Task impairments (APM Task Score > 1.96) and those without (APM Task Score < 1.96). For this analysis, APM Task Scores were binarized to an impaired and unimpaired group, given that higher scores are associated with worse performance, but do not necessarily mean that performance falls outside of the normal range of control behaviour (unless the score is > 1.96). This analysis was therefore important for determining the white matter disconnection specifically associated with APM Task impairments and not just a worse score that still may be within the realm of normal for control participants. General Linear Models (GLM) were performed on the disconnectome maps (*see* *Materials and Methods*: *Disconnectome maps*) between the two groups, using the ‘Randomise’ tool in FSL [61] (https://fsl.fmrib.ox.ac.uk/fsl/fslwiki/Randomise). This tool allows for non-parametric permutation inference on neuroimaging data. Grey matter lesion volume was included in the GLM as a covariate, to account for damage to non-white matter structures (an additional uncontrolled analysis was also performed). Threshold-Free Cluster-Enhancement was used to address the multiple comparisons problem with neuroimaging data and enhance cluster-like structures within the data [79]. Similar methods have been applied previously [47]. The data was subject to 4000 permutations and corrected for family-wise error rate (p>0.95). Clusters with a corrected p-value of less than 0.05 were deemed significant. Voxel-wise lesion/disconnectome analysis can suffer from statistical power limitations and overshoot [80], whereby statistical signal can cover an unreasonable number of voxels. As such, a post-hoc analysis was also performed, considering voxels with a higher statistical peak (p<0.001) to increase the confidence that those regions play a causal role in proprioception.

## Results

### Demographics

**Fig 2:**
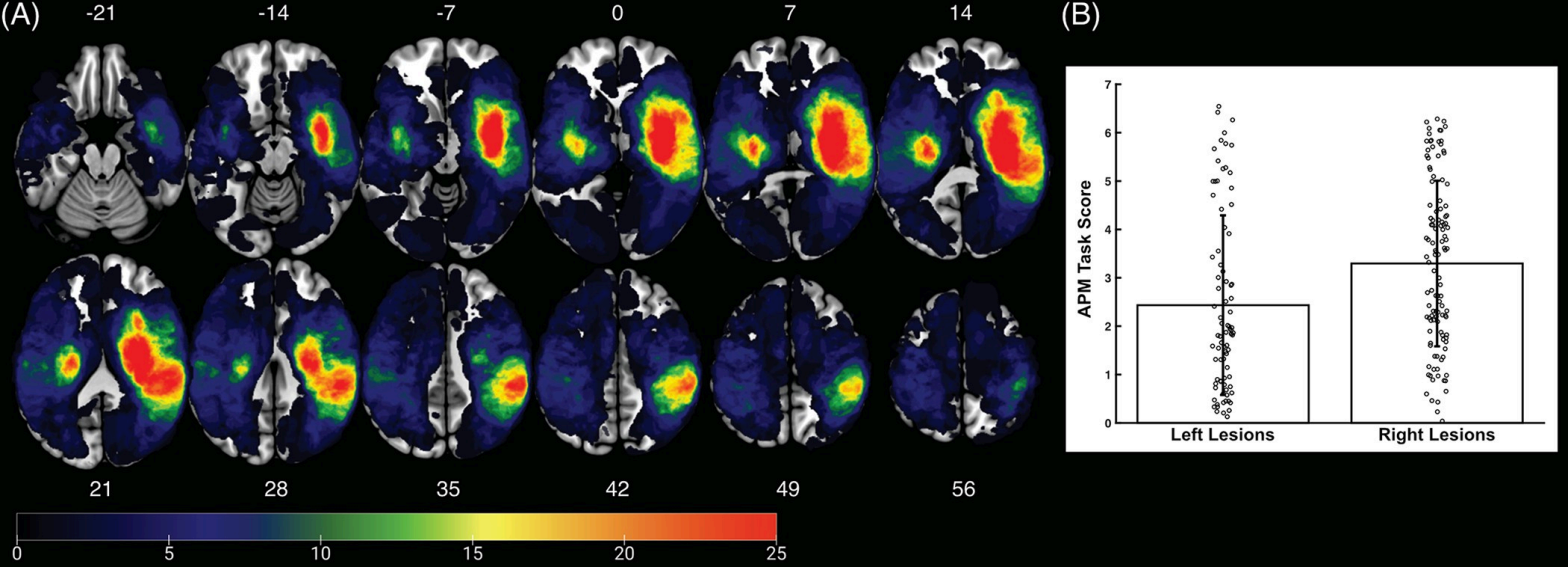
Lesion overlap. (A) Lesion overlap map for all 203 participants. Lesions presented in neurologic convention. Numbers above (top) and below (bottom) each slice indicate the axial MNI coordinate. (B) Mean Arm Position Matching (APM) Task Scores for the left hemisphere lesion group (n = 85) and right hemisphere lesion group (n = 118). Open circles are individual data points for each participant. Black bars indicate the standard deviation around the mean for each group.

Participant demographics are presented in Table 2. In total, 203 participants were included in the study (128 Male, 75 Female; Mean age 62.2 ± 14.4 years; 118 right lesions, 85 left lesions). Fig 2A displays the lesion overlap for all participants. Mean total lesion volume across all participants was 30.5 ± 39.3 cc. Mean grey matter lesion volume across all participants was 11.0 ± 15.2 cc. Impairments on the APM task were apparent for 127 participants, with 76 participants unimpaired on the task.

**Table 2:** Participant demographics.

**Age (years)**	62.2 ± 14.2
**Sex n[M,F]**	[128, 75]
**Lesioned Hemisphere n[R,L]**	[118, 85]
**Total Lesion Volume (cc)**	30.5 ± 39.3
**Grey Matter Lesion Volume (cc)**	11.0 ± 15.2
**TLT n[0,1,2,3]** ^**a**^	[97, 51, 39, 15]
**CMSA Affected Arm n[1,2,3,4,5,6,7]** ^**a**^	[17, 19, 25, 12, 51, 33, 45]
**CMSA Less Affected Arm n[1,2,3,4,5,6,7]** ^**a**^	[0, 1, 0, 0, 3, 32, 166]
**BIT (median [range])** ^**a**^	140.5 [51–146]
**FIM (median [range])** ^**a**^	102.5 [35–126]

Data presented are mean ± standard deviation, unless otherwise stated. TLT = Thumb Localization Test, CMSA = Chedoke McMaster Stroke Assessment, BIT = Behavioural Inattention Test, FIM = Functional Independence Measure. For the TLT and CMSA, the numbers in square parentheses indicate the number of participants who obtained each score. For the BIT and FIM, the numbers in square parentheses indicate the range.

^a^ Clinical data missing for one participant.

### Comparison of left vs right lesions

The first analysis compared APM Task Scores between participants with left vs right lesions (Fig 2B). When accounting for lesion volume, APM Task Scores were significantly greater/worse for participants with right lesions (F = 7.798, p = 0.00574). Ipsilesional motor impairments were mild across the sample, with respect to CMSA scores. There was no significant association between ipsilesional motor impairments and lesioned hemisphere (χ^2^ = 0.844, p = 0.358; left = 15.3% of sample, right = 20.3% of sample). This is important considering ipsilesional motor impairments could impact a participant’s ability to perform the APM Task, thus ruling out these impairments as a potential explanation as to hemispheric differences in APM performance.

### Multivariate linear regression between white matter tract damage and APM task scores

#### Effect of white matter tract lesion load

**Fig 3:**
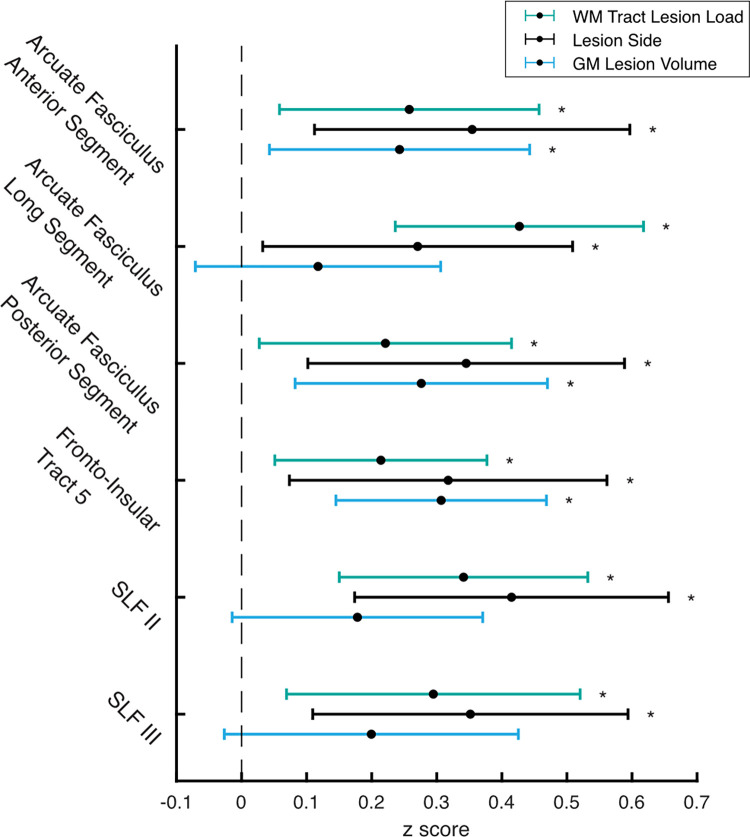
Multivariate regression results. Coefficient estimates and 95% confidence intervals for white matter tract lesion load (green), lesion side (black) and grey matter lesion volume (blue). Coefficients are only displayed for the white matter tracts where significant relationships occurred between the tract lesion load and Arm Position Matching (APM) Task Scores. * indicates a significant coefficient estimate at a 5% false discovery rate. SLF = Superior Longitudinal Fasciculus.

Fig 3 displays the tracts with a significant association between the tract lesion load and APM Task Scores. The effect of increasing damage, independent of the extent of grey matter damage and regardless of lesion side, was significant for the: Arcuate Fasciculus Anterior Segment, Arcuate Fasciculus Long Segment, Arcuate Fasciculus Posterior Segment, Fronto-Insular Tract 5, SLF II and SLF III (Fig 3). Of the other 17 tracts evaluated, none showed significant relationships between the tract lesion load and APM Task Scores (S1 Fig). Across all regression models, R^2^ values ranged from 0.24–0.31, equating to effect sizes (f^2^) of 0.32–0.44. A subsequent power analysis demonstrated the analysis to be fully powered (100%). Unsurprisingly, in the uncontrolled analysis (without covarying for grey matter lesion volume), many more tracts displayed apparent associations with APM Task Score. All tracts were deemed significant, except for the: Cingulum, Frontal Commissure and Frontal Superior Longitudinal U-tract (S2 Fig).

#### Effect of lesion side

Lesion side was a significant predictor of APM Task Scores in the multivariate regression analysis, for all 23 tracts (S3 Fig). In these tracts, APM Task Scores were significantly greater (indicating worse performance) for participants with right lesions. This was true regardless of the lesion load to each tract, or grey matter lesion volume, in addition to the uncontrolled analysis (S4 Fig)

#### Effect of grey matter lesion volume

Grey matter lesion volume was a significant predictor of APM Task Scores in the multivariate regression analysis for each tract, *except* for the: Arcuate Fasciculus Long Segment, Corpus Callosum, SLF II and SLF III (S5 Fig). When controlling for the lesion load to each tract and irrespective of lesion side, increasing grey matter lesion volume had the effect of increasing/worsening APM Task Score.

### Principal component regression

An additional principal component regression was performed to address concerns of collinearity, with respect to the lesion load to each tract. PCA reduces the dimensionality of the predictor variables being tested for relationships with APM Task Scores. The initial PCA demonstrated that nine components explained 95.6% of variance in the predictor variables (S6 Fig). The subsequent multivariate analysis demonstrated significant relationships with APM Task Scores for four of these nine principal components (Table 3). Significant positive relationships were observed for PC1 (p = 2.717 x10^-15^), PC5 (p = 0.003) and PC7 (p = 0.014), with a significant negative relationship observed for PC2 (p = 2.744 x10^-4^).

**Table 3:** Principal component regression results.

	Estimate	SE	t-stat	p-value
**Intercept**	6.299 x10^-16^	0.058	1.078 x10^-14^	1
**PC1**	0.150	0.018	8.232	**2.717 x10** ^**-14**^
**PC2**	-0.010	0.027	-3.707	**2.744 x10** ^**-4**^
**PC3**	-0.036	0.036	-1.005	0.316
**PC4**	-0.008	0.038	-0.209	0.835
**PC5**	0.183	0.061	3.007	**0.003**
**PC6**	0.001	0.072	0.019	0.985
**PC7**	0.209	0.085	2.469	**0.014**
**PC8**	0.023	0.094	0.243	0.808
**PC9**	0.080	0.101	0.796	0.427

Principal Component analysis results obtained from the regression analysis between principal component scores for the first 9 principal components and Arm Position Matching Task Scores. The first 9 principal components explained 95% of the variance in white matter lesion load across the sample. Bolded p-values indicate those with significant relationships which survived FDR (5%) correction.

Table 4 highlights the factor loadings of the three principal components with significant relationships with APM Task Score (PC1, PC2, PC5 and PC7). Factor loadings for all other PCs are presented in S1 Table.

**Table 4:** Principal component analysis loadings.

	PC1	PC2	PC5	PC7
**Variance Explained (%)**	***43*.*2***	***19*.*7***	***3*.*8***	***2*.*0***
**Eigenvalue**	***10*.*4***	***4*.*7***	***0*.*9***	***0*.*5***
**Corpus Callosum**	0.29	0.02	-0.05	-0.01
**Grey Matter Lesion Volume**	0.28	-0.13	-0.04	-0.17
**SLF III**	0.25	-0.18	-0.06	0.03
**Arcuate Fasciculus Long Segment**	0.24	-0.21	0.13	0.40
**Fronto Insular Tract 5**	0.23	-0.15	0.01	0.02
**SLF II**	0.23	-0.08	0.14	0.12
**Arcuate Fasciculus Anterior Segment**	0.23	-0.19	-0.01	-0.02
**Frontal Aslant**	0.23	0.14	-0.41	-0.09
**Fronto Insular Tract 4**	0.23	-0.10	-0.23	-0.14
**Fronto Striatal**	0.22	0.19	-0.08	0.05
**IFOF**	0.22	-0.15	-0.35	-0.14
**Anterior Thalamic Projections**	0.21	0.27	-0.09	0.01
**CST**	0.21	0.24	0.18	0.34
**Pons**	0.20	0.26	0.10	0.29
**Arcuate Fasciculus Posterior Segment**	0.19	-0.24	0.21	0.33
**Anterior Commissure**	0.18	-0.06	0.37	-0.21
**Frontal Commissure**	0.17	0.35	-0.03	-0.12
**Fornix**	0.16	-0.08	0.48	-0.38
**Hand Inferior U Tract**	0.15	-0.10	0.23	-0.38
**ILF**	0.15	-0.20	-0.15	-0.07
**SLF I**	0.14	0.30	0.08	0.12
**Frontal Superior Longitudinal**	0.13	0.33	0.07	-0.13
**Cingulum**	0.12	0.31	0.01	-0.22
**Optic Radiations**	0.12	-0.15	-0.26	0.05

The amount of variance explained (%), eigenvalues and principal component loading factors for the four principal components which displayed significant relationships with APM Task Score. Principal components are ordered according to the amount of variance explained. Variables are ordered according to their weighting on principal component 1. PC = principal component, SLF = superior longitudinal fasciculus, IFOF = inferior fronto occipital fasciculus, CST = corticospinal tract, ILF = inferior longitudinal fasciculus.

For the first component (PC1), factor loadings were evenly spread across numerous predictor variables (Table 4). PC1 was comprised, equally, of commissural white matter tracts (Corpus Callosum), grey matter lesion volume as well as perisylvian white matter tracts (SLF III, Arcuate Fasciculus Long Segment, SLF II, Arcuate Fasciculus Anterior Segment) volume. The Frontal Aslant, Fronto Insular U Tracts 4 and 5 and Inferior Fronto-Occipital Fasciculus (IFOF) also contributed to PC1 with positive factor loadings. The fifth and seventh components (PC5 and PC7) also showed positive relationships with APM Task Scores, however they only explained a combined 5.8% of variance.

The second component (PC2) explained 19.7% of variance and was negatively associated with APM Task Scores (Table 4). Factors which contributed most to this component comprised of the Frontal Commissure, Frontal Superior Longitudinal U Tract, Cingulum, SLF I and Anterior Thalamic Projections.

### White matter disconnection and APM task impairments

**Fig 4:**
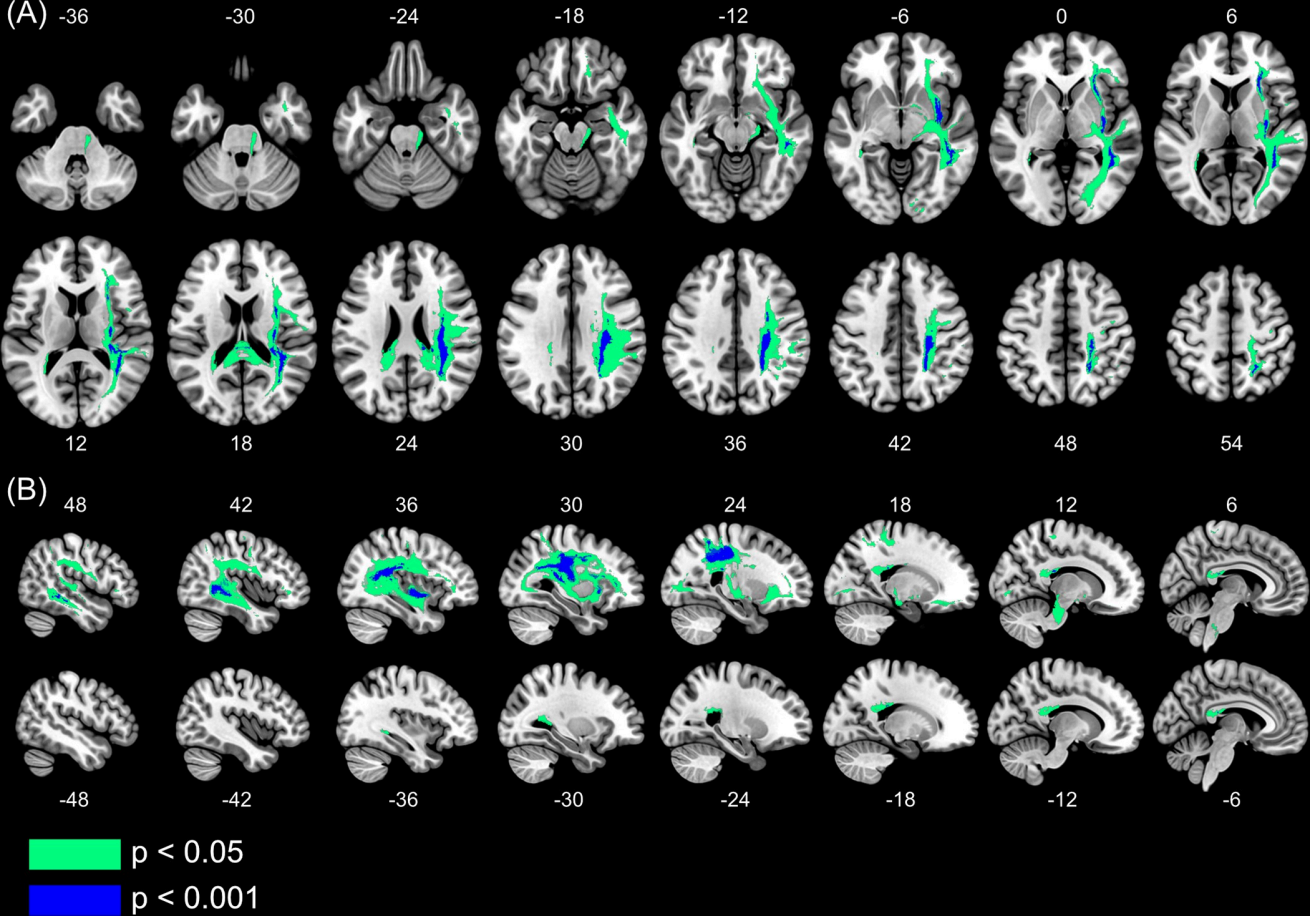
Disconnectome analysis results. Voxels with a greater probability of disconnection in participants with Arm Position Matching (APM) Task impairments compared to participants without APM Task impairments. Voxels in green are significant at p<0.05. Voxels in blue are significant at a higher statistical peak of p<0.001. (A) Axial view. Images are presented in neurological convention. Numbers above (top) and below (bottom) each slice indicate the axial MNI coordinate. (B) Sagittal view. Right and left hemisphere are shown. Numbers above (top) and below (bottom) each slice indicate the sagittal MNI coordinate. Structures where disconnection was associated with APM task impairments include: Medial Lemniscus, Cerebral Peduncles, Internal Capsule, Corona Radiata, S1 white matter, Corpus Callosum, Arcuate Fasciculus, SLF II, SLF III, IFOF, External Capsule and Optic Radiations.

The final analysis compared the disconnectome maps (*see* *Materials and Methods*) between those with and without APM task impairments, to assess voxel-wise differences in disconnection throughout the brain. That is, voxels within the white matter which, when disconnected, result in impaired APM task performance. Fig 4 displays voxels within the white matter with significantly greater probability of disconnection in those with impairments on the APM task, than those without impairments on the APM task. These clusters of voxels were distributed across key structures in the right hemisphere, with a statistical peak (p<0.001) around the Arcuate Fasciculus, SLF, Corona Radiata (particularly underlying S1), External Capsule and IFOF. Other significant (p<0.05) white matter structures included: Medial Lemniscus, Cerebral Peduncles, Internal Capsule, Corpus Callosum, Inferior Longitudinal Fasciculus (ILF) and Optic Radiations. The analysis, without controlling for grey matter lesion volume, revealed widespread white matter disconnection throughout both hemispheres to be associated with APM task impairments (S7 Fig).

## Discussion

This study investigated the white matter disconnections associated with impaired proprioception post-stroke. It highlights distributed white matter correlates of poor proprioception post-stroke, with particularly important roles of the perisylvian (Arcuate Fasciculus, Superior Longitudinal Fasciculus), fronto-insular (Fronto Insular Tract 5) and commissural (Corpus Callosum) white matter, when performing position matching tasks with the upper limb. It also provides substantial evidence that proprioception is worse following right hemisphere damage after stroke.

**Fig 5:**
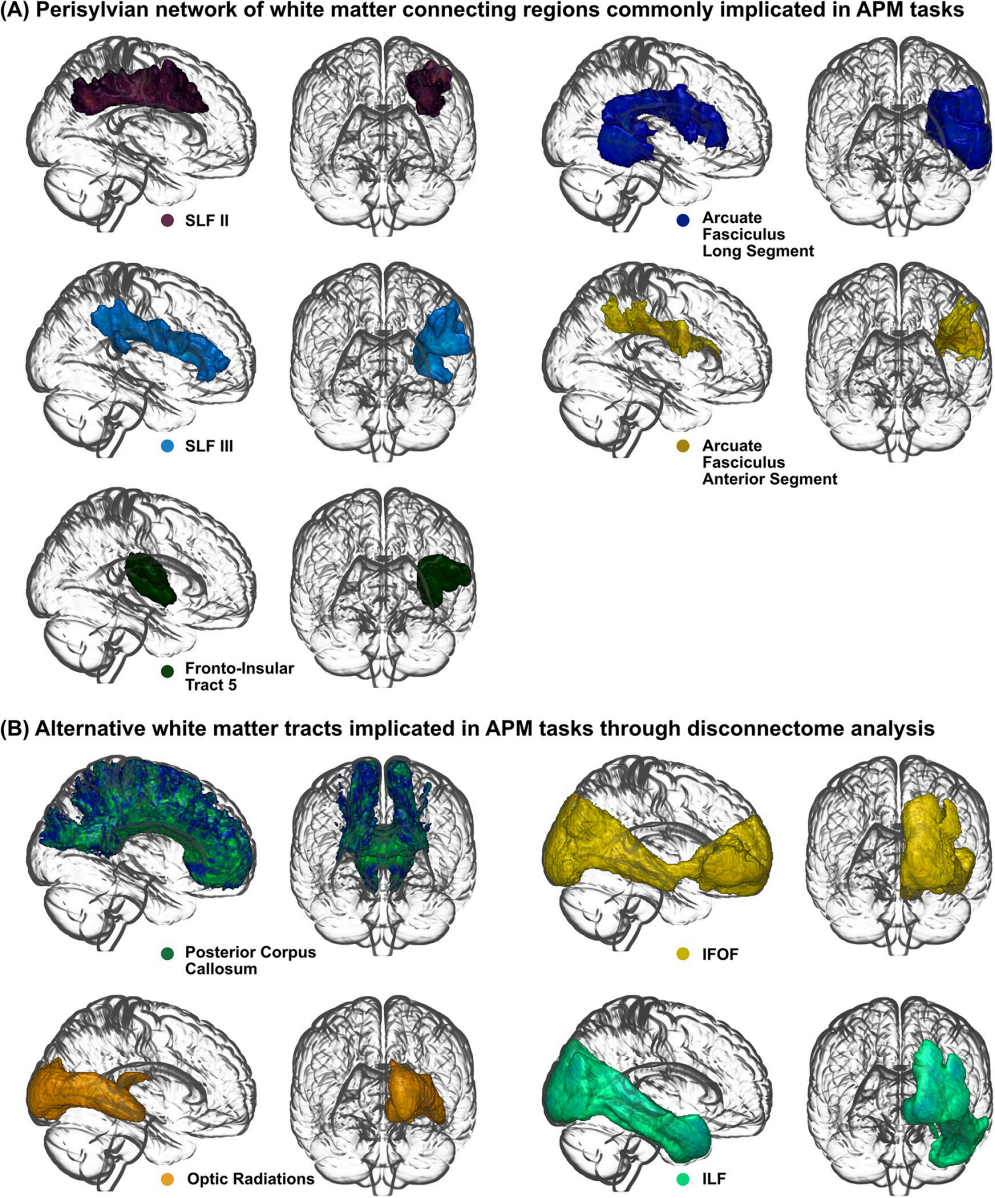
White matter network in APM tasks. (A) White matter tracts connecting cortical regions previously implicated in proprioceptive tasks, in particular, Arm Position Matching (APM) tasks. White matter tracts include: Superior Longitudinal Fasciculus II (SLF II; Purple), Superior Longitudinal Fasciculus III (SLF III; Light Blue), Arcuate Fasciculus Long Segment (Dark Blue), Arcuate Fasciculus Anterior Segment (Yellow), and Fronto Insular Tract 5 (Dark Green). (B) Alternative white matter tracts, implicated in APM task impairments, as observed by disconnectome analysis. White matter tracts include: Posterior Corpus Callosum (Green/Blue), Inferior Fronto Occipital Fasciculus (IFOF; Yellow), Optic Radiations (Orange) and, Inferior Longitudinal Fasciculus (ILF; Light Green).

Compared to the grey matter correlates of proprioception, the white matter has received far less attention. While evidence points towards a wide network of cortical regions being involved in proprioceptive processing, this study highlights a white matter framework which may underpin many perisylvian regions that have previously linked with proprioception (Fig 5). Using several techniques, many cortical regions have been postulated to be responsible for proprioceptive processing in the brain. Regions include, but are not limited to: S1, supramarginal gyrus, angular gyrus, insula, superior parietal lobe, superior temporal gyrus, Heschl’s gyrus and premotor cortex [12, 13, 18, 20, 23, 24]. The findings of the present study suggest that many cortico-cortical white matter structures, connecting important proprioceptive grey matter regions, are also important anatomical structures proprioception, with their disconnection correlates of proprioceptive impairment post-stroke. By accounting for damage to the grey matter in each analysis, white matter disconnection proved an important correlate of proprioceptive impairment, post-stroke, independent of grey matter damage.

The current study is not the first to associate the perisylvian white matter with proprioception, demonstrating an important role of the arcuate fasciculus and SLF (II and III) (Figs 3 and 4) [18–20, 26]. While the arcuate fasciculus and SLF are predominantly considered as language pathways in the left hemisphere [81], previous functional imaging work has shown activation of inferior parietal and premotor regions, connected by the arcuate fasciculus and SLF, are integral to accurate performance of position matching tasks [17, 24] and kinaesthetic awareness [27]. Further work from our lab has also shown microstructural changes (fractional anisotropy) in the arcuate fasciculus and SLF to be associated with poorer APM performance after stroke [26]. It is plausible that the arcuate fasciculus and SLF are also responsible for transferring proprioceptive information between parietal and frontal regions when performing proprioceptive tasks, particularly in the right hemisphere [14, 27, 82], with the left hemisphere being specialized for language functions [81].

Furthermore, the findings of this study advance our understanding that the communication between the insula and somatosensory cortex is crucial for the proprioceptive sense. While previous literature has linked the posterior insula with proprioception, bodily awareness and spatial neglect [83–87], greater lesion load to the fronto-insular tract 5, a small white matter tract connecting the somatosensory and posterior insula [53], was also a significant correlate of poorer APM Task performance (Fig 3).

Lesion load metrics are, however, limited when considering white matter disconnection, due to: 1) the multi-directional nature of white matter fibres at a given voxel and 2) the inherent assumption that increased white matter lesion load results in worse disconnection symptom severity [54, 55]. As a result, we adopted a complementary voxel-based disconnectome analysis. This approach is advantageous as it assesses the impact that lesions have on the disconnection in voxels of the white matter, distant from the lesion itself, and does not carry the assumption that increasing white matter lesion load tract increases symptom severity. Interestingly, this additional analysis revealed alternative white matter tracts which, when disconnected, resulted in impaired proprioception post-stroke (Figs 4 and 5B). These white matter tracts included, corpus callosum (posterior), IFOF, ILF and, to a lesser extent, optic radiations. Interestingly, the majority of these white matter tracts have not been associated with proprioceptive function previously.

Of the alternative white matter tracts implicated in the voxel-wise disconnectome analysis, the corpus callosum was perhaps the least surprising tract to see associated with impaired proprioception. While other anatomical investigations of proprioception have not implicated the importance of the corpus callosum, these studies they often relied upon tendon vibration and passive movement of a single limb only [11–13]. By contrast, the APM Task used to assess proprioception in the present study was a bilateral task. The somatosensory cortices are connected bilaterally through the posterior corpus callosum [88–90], which has been demonstrated to be important for the interhemispheric transfer of sensory information [91–93]. This functional role likely explains the association between posterior corpus callosum disconnection and impairments on the APM task.

Perhaps the most surprising disconnectome finding, was the high statistical peak in the IFOF, with a smaller statistical relationship found for the ILF and optic radiations (Fig 4). While it is plausible that these findings could be an overshoot in the interpretation of lesion/disconnection-symptom mapping [80], each also has viable explanations as to its potential implications in the APM Task. The IFOF connects occipital, parietal, temporal and frontal regions [94–96], with high amounts of connectivity between the superior parietal lobule and premotor and prefrontal areas, through the IFOF [97]. It is therefore possible that, like the arcuate and SLF above, the IFOF functions more generally in the transfer of information from parietal, temporal and occipital regions to the frontal lobe in sensorimotor tasks [96].

The lingual branch of the ILF connects the lingual and middle temporal gyri [98] and has been functionally associated with visual memory [99, 100], while the optic radiations relay visual information from the lateral geniculate nucleus to the visual cortex. Considering the APM task was performed with vision of the limbs occluded, one possibility is that during the APM task, participants generate visual representations of the positions of their limbs using mental and motor imagery. Both processes are known to activate similar regions to visual and motor tasks and might give insight into the association of these white matter tracts and APM task impairments [101–103].

Previous work, in smaller samples, has suggested that proprioception might be lateralised to the right hemisphere [12, 14, 72, 73, 104, 105]. The present study supports this notion in two ways. Firstly, APM Task performance was worse in those with right hemisphere lesions compared to left hemisphere lesions (Fig 2). Secondly, disconnection of white matter in the right, but not left, hemisphere was associated with APM Task impairments (Fig 4). Together these findings support the right hemisphere specialization for proprioceptive processing.

Previous literature has used alternative methods of assessing upper-limb proprioception such as two-alternative forced choice paradigms [106–108] and passive matching tasks using the same arm [109]. These methods take considerable time to perform and are challenging in the face of motor impairments, respectively. The APM Task used in this study only takes around two and a half minutes to perform. Furthermore, bilateral position matching tasks are also useful to assess proprioception following stroke, as they require only passive movement of the affected arm. As such, participants with severe contralesional motor impairments can still be easily assessed. While bilateral tasks are more complex, requiring proprioception processing to occur between the two limbs, and consequently hemispheres, many activities of daily living rely upon this coordination of proprioceptive processing between the two limbs. It must be considered that the nature of the APM Task might explain some of the anatomical structures implicated in these findings.

Within our supplementary analysis we controlled for hemispatial neglect, however there are also other neuropsychological phenomenon such as body awareness deficits, pathological embodiment, somatoparaphrenia and anosognosia for hemiplegia, that are somewhat related to proprioception and share similar neural substrates to those found in this study (such as the arcuate fasciculus and superior longitudinal fasciculus), which are also associated with right hemisphere lesions [47, 110–113]. While these phenomenon were not recognized by the clinicians treating, nor the ones assessing the participants in the current study, our research team did not formally test for them and it would therefore we must recognize there is a possibility these rarer, alternative disorders could have been present in some participants and may impact our findings.

### Limitations

Like all studies, the current work has its limitations. As previously mentioned, white matter lesion load metrics rely on the underlying assumption that *increasing* lesion loads result in worse impairments. In this regard, the disconnection analysis is important as it does not rely on this assumption, rather identifying disconnected voxels/clusters that relate to impairments, irrespective of the amount of damage. Voxel-based metrics are, however, susceptible to distortions in anatomical findings [80, 114, 115], through an overshoot in statistically significant signal. While permutation testing and post-hoc analyses, at a higher statistical threshold, were used to try to reduce this likelihood, it is impossible to rule out the potential for this overshoot in the interpretations of these findings.

Unfortunately, anatomical variables following stroke, such as regional lesion loads and lesion volume, are often collinear and highly correlated with one another. This can lead to confounding and suppression effects [116, 117], making the effect of each individual variable difficult to interpret. Consequently, a principal component regression was used to address concerns with collinearity with regards to white matter tract lesion. While scores from the first principal component were significantly associated with APM Task Scores, factor loadings varied very little, ranging from 0.29 to 0.12 for PC1. As such, some caution should be taken when interpreting these results. Furthermore, grey matter lesion volume was included as a predictor variable in the PCA. As such, the loading for each white matter tract are only relative to, and not independent of grey matter lesion volume.

Despite demonstrating evidence of a greater importance for the right hemisphere and right hemisphere white matter in proprioception, the inclusion criteria limited entry into the study for participants with aphasia, who likely had larger left hemisphere strokes. As such, there was an imbalance in the number of participants and lesion volumes between those with left and right hemisphere lesions. Although measures were taken to control for lesion volume in the analysis, these differences make right vs left hemisphere comparisons challenging.

## Conclusion

This study demonstrated how lesions to, and disconnections of, key white matter structures impact performance on a proprioceptive Arm Position Matching task after stroke. In doing so, it furthers our understanding of the white matter that is critical for proprioception, relative to grey matter damage post-stroke. While many tracts underpin a perisylvian network of cortical regions that are important for proprioception, it also revealed that many white matter tracts, connecting parietal, temporal and frontal regions, despite not traditionally being viewed as proprioceptive tracts, may all be involved in carrying sensory information between cortical regions implicated in proprioceptive tasks.

## Supporting information

S1 Fig White matter tract lesion load coefficient estimates. Coefficient estimates and 95% confidence intervals for the relationship between white matter tract lesion load and Arm Position Matching (APM) Task Scores for all white matter tracts tested. * indicates a significant coefficient estimate (5% false discovery rate).
(PDF)

S2 Fig White matter tract lesion load coefficient estimates (analysis uncontrolled for grey matter lesion volume). Coefficient estimates and 95% confidence intervals for the relationship between the white matter tract lesion load and Arm Position Matching (APM) Task Scores for all white matter tracts tested in the uncontrolled analysis, without the influence of grey-matter lesion volume. * indicates a significant coefficient estimate (5% false discovery rate).
(PDF)

S3 Fig Lesion side coefficient estimates. Coefficient estimates and 95% confidence intervals for the relationship between lesion side and Arm Position Matching (APM) Task Scores for all white matter tracts tested. * indicates a significant coefficient estimate (5% false discovery rate).
(PDF)

S4 Fig Lesion side coefficient estimates (Analysis uncontrolled for grey matter lesion volume). Coefficient estimates and 95% confidence intervals for the relationship between lesion side and Arm Position Matching (APM) Task Scores for all white matter tracts tested in the uncontrolled analysis, without the influence of grey-matter lesion volume. * indicates a significant coefficient estimate (5% false discovery rate).
(PDF)

S5 Fig Grey matter lesion volume coefficient estimates. Coefficient estimates and 95% confidence intervals for the relationship between grey matter lesion volume and Arm Position Matching (APM) Task Scores for all white matter tracts tested. * indicates a significant coefficient estimate (5% false discovery rate).
(PDF)

S6 Fig Variance explained. The percentage of variance explained for the first ten components of the principal component analysis performed on the extent of damage to each white matter tract. Dashed line indicates 95% of variance explained. As can be seen, nine principal components explained 95% of the total variance (dashed horizontal line).
(PDF)

S7 Fig Disconnectome analysis (analysis uncontrolled for grey matter lesion volume). Voxels with a greater probability of disconnection in participants with Arm Position Matching (APM) Task impairments compared to participants without APM Task impairments. A) Axial view. Images are presented in neurological convention. Numbers above (top) and below (bottom) each slice indicate the axial MNI coordinate. B) Sagittal view. Right (top) and left (bottom) hemisphere are shown. Numbers above (top) and below (bottom) each slice indicate the sagittal MNI coordinate. Note: analysis excludes grey matter lesion volume as a covariate.
(PDF)

S1 Table Principal component analysis loadings. The amount of variance explained (%), eigenvalues and principal component loading factors for the lesion load to each white matter tract, on the nine principal components which explained 95% of the total variance. Principal components are ordered according to the amount of variance explained. Variables are ordered according to their weighting on principal component 1. PC = principal component, SLF = superior longitudinal fasciculus, IFOF = inferior fronto occipital fasciculus, CST = corticospinal tract, ILF = inferior longitudinal fasciculus.
(PDF)

S2 Table Principal component regression results (analysis uncontrolled for grey matter lesion volume). Principal Component Regression analysis results obtained from the regression analysis between principal component scores for the first 9 principal components and Arm Position Matching Task Scores. The first 9 principal components explained 95% of the variance in white matter lesion load across the sample. Bolded p-values indicate those with significant relationships which survived FDR (5%) correction. Note: Analysis excludes grey matter lesion volume.
(PDF)

S3 Table Principal component analysis loadings (analysis uncontrolled for grey matter lesion volume). The amount of variance explained (%), eigenvalues and principal component loading factors for the lesion load to each white matter tract, on the three principal components which displayed significant relationships with APM Task Score. Principal components are ordered according to the amount of variance explained. Variables are ordered according to their weighting on principal component 1. PC = principal component, SLF = superior longitudinal fasciculus, IFOF = inferior fronto occipital fasciculus, CST = corticospinal tract, ILF = inferior longitudinal fasciculus. Note: Analysis excluded grey matter lesion volume.
(PDF)

S1 Dataset Participant behavioural data. Individual data for each of the 203 participants. Order of participant data is the same as the participant disconnectome maps in S1 File.
(CSV)

S1 File Participant disconnectome maps. Individual disconnectome maps for each of the 203 participants in a 4D.nii file. Order of disconnectome maps are the same as the participant behavioural data in S1 Dataset.
(GZ)
