# Supplementary material for: White matter disconnection impacts proprioception post-stroke
Source: PLoS One. 2024 Sep 12;19(9):e0310312. doi: 10.1371/journal.pone.0310312 (PMC11392420; doi:10.1371/journal.pone.0310312)

S2 Fig. White matter tract lesion load coefficient estimates (Analysis uncontrolled for grey matter lesion volume) - Coefficient estimates and 95% confidence intervals for the relationship between the white matter tract lesion load and Arm Position Matching (APM) Task Scores for all white matter tracts tested in the uncontrolled analysis, without the influence of grey-matter lesion volume. * indicates a significant coefficient estimate (5% False Discovery Rate).

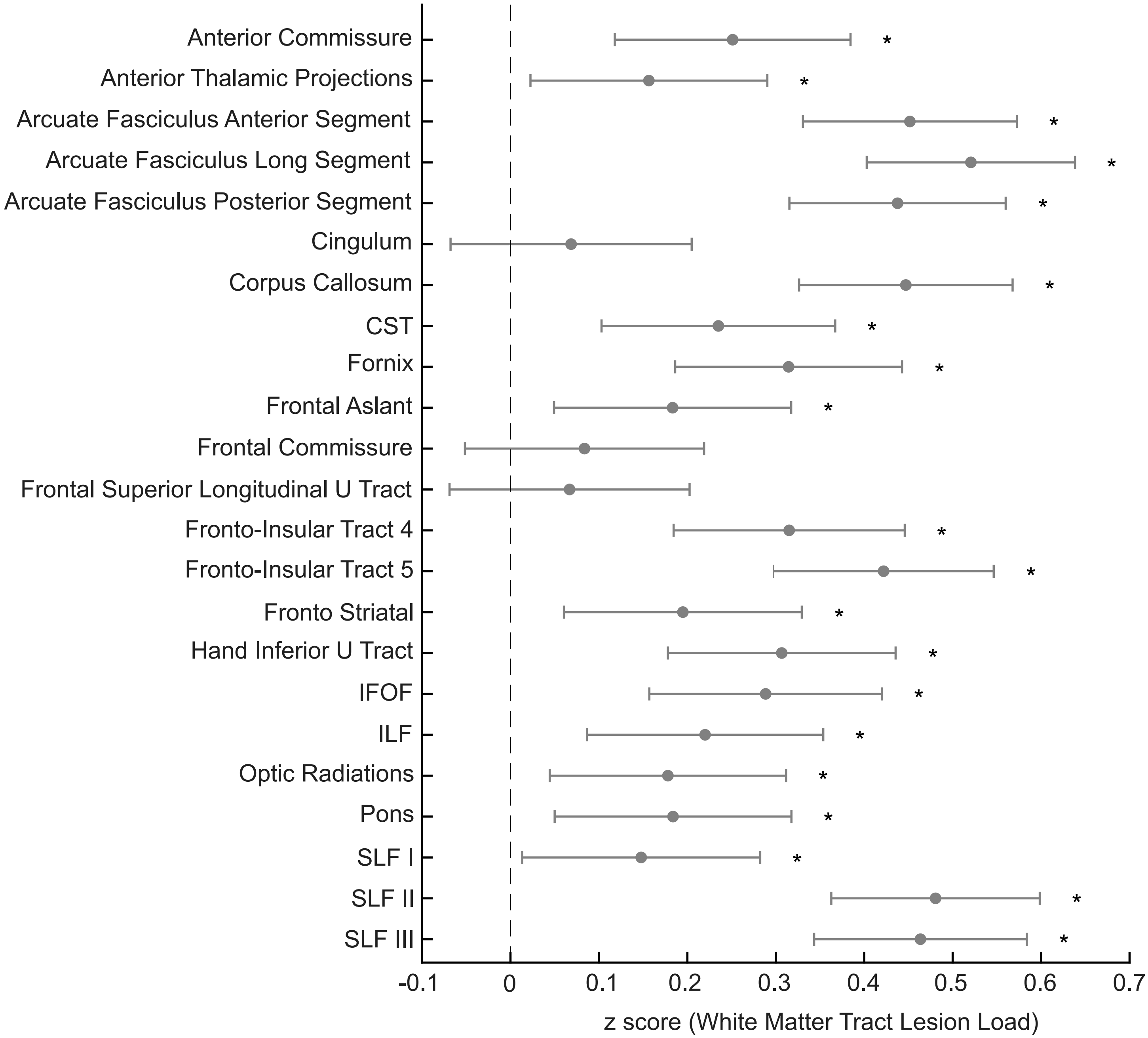

Supplement: S2 Fig — Coefficient estimates and 95% confidence intervals for the relationship between the white matter tract lesion load and Arm Position Matching (APM) Task Scores for all white matter tracts tested in the uncontrolled analysis, without the influence of grey-matter lesion volume. * indicates a significant coefficient estimate (5% false discovery rate). (PDF) [file pone.0310312.s002.pdf]
