# Supplementary material for: White matter disconnection impacts proprioception post-stroke
Source: PLoS One. 2024 Sep 12;19(9):e0310312. doi: 10.1371/journal.pone.0310312 (PMC11392420; doi:10.1371/journal.pone.0310312)

S4 Fig. Lesion side coefficient estimates (Analysis uncontrolled for grey matter lesion volume). Coefficient estimates and 95% confidence intervals for the relationship between lesion side and Arm Position Matching (APM) Task Scores for all white matter tracts tested in the uncontrolled analysis, without the influence of grey-matter lesion volume. * indicates a significant coefficient estimate (5% False Discovery Rate).

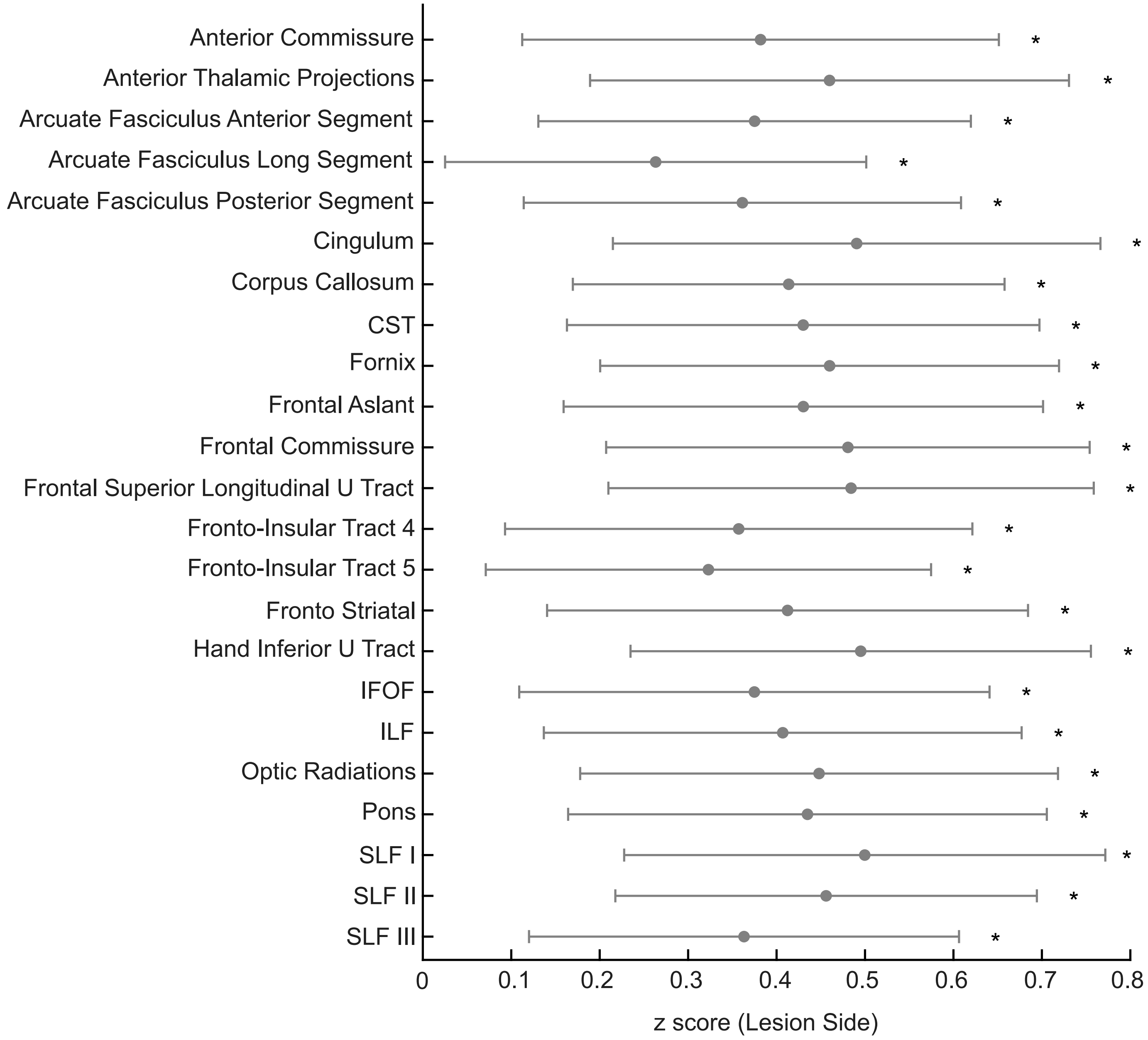

Supplement: S4 Fig — Coefficient estimates and 95% confidence intervals for the relationship between lesion side and Arm Position Matching (APM) Task Scores for all white matter tracts tested in the uncontrolled analysis, without the influence of grey-matter lesion volume. * indicates a significant coefficient estimate (5% false discovery rate). (PDF) [file pone.0310312.s004.pdf]
