# Supplementary material for: White matter disconnection impacts proprioception post-stroke
Source: PLoS One. 2024 Sep 12;19(9):e0310312. doi: 10.1371/journal.pone.0310312 (PMC11392420; doi:10.1371/journal.pone.0310312)

S5 Fig. Grey matter lesion volume coefficient estimates. Coefficient estimates and 95% confidence intervals for the relationship between grey matter lesion volume and Arm Position Matching (APM) Task Scores for all white matter tracts tested. * indicates a significant coefficient estimate (5% False Discovery Rate).

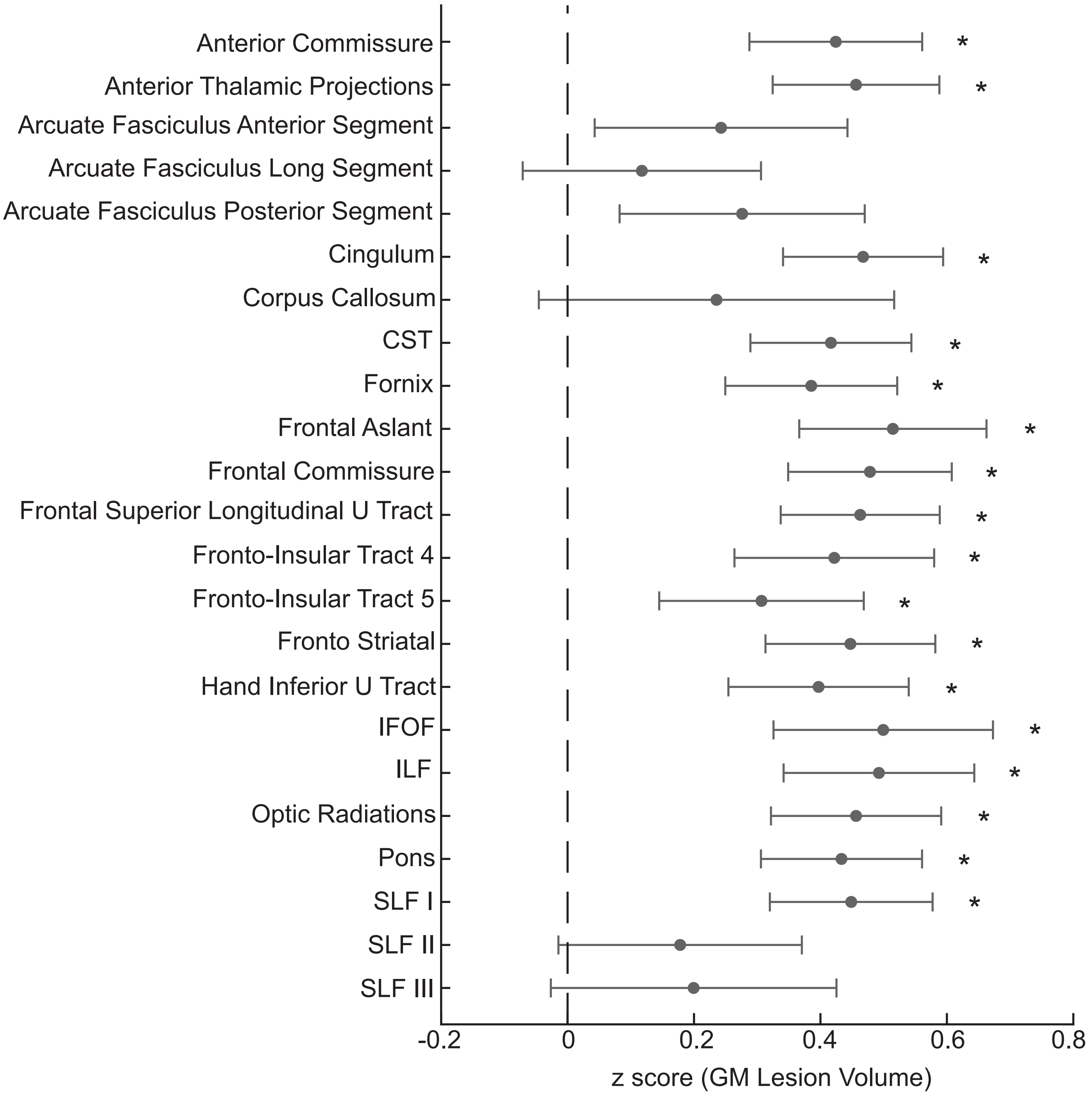

Supplement: S5 Fig — Coefficient estimates and 95% confidence intervals for the relationship between grey matter lesion volume and Arm Position Matching (APM) Task Scores for all white matter tracts tested. * indicates a significant coefficient estimate (5% false discovery rate). (PDF) [file pone.0310312.s005.pdf]
