# Supplementary material for: White matter disconnection impacts proprioception post-stroke
Source: PLoS One. 2024 Sep 12;19(9):e0310312. doi: 10.1371/journal.pone.0310312 (PMC11392420; doi:10.1371/journal.pone.0310312)

S6 Fig. Variance explained. The percentage of variance explained for the first ten components of the principal component analysis performed on the extent of damage to each white matter tract. Dashed line indicates 95% of variance explained. As can be seen, nine principal components explained 95% of the total variance (dashed horizontal line).

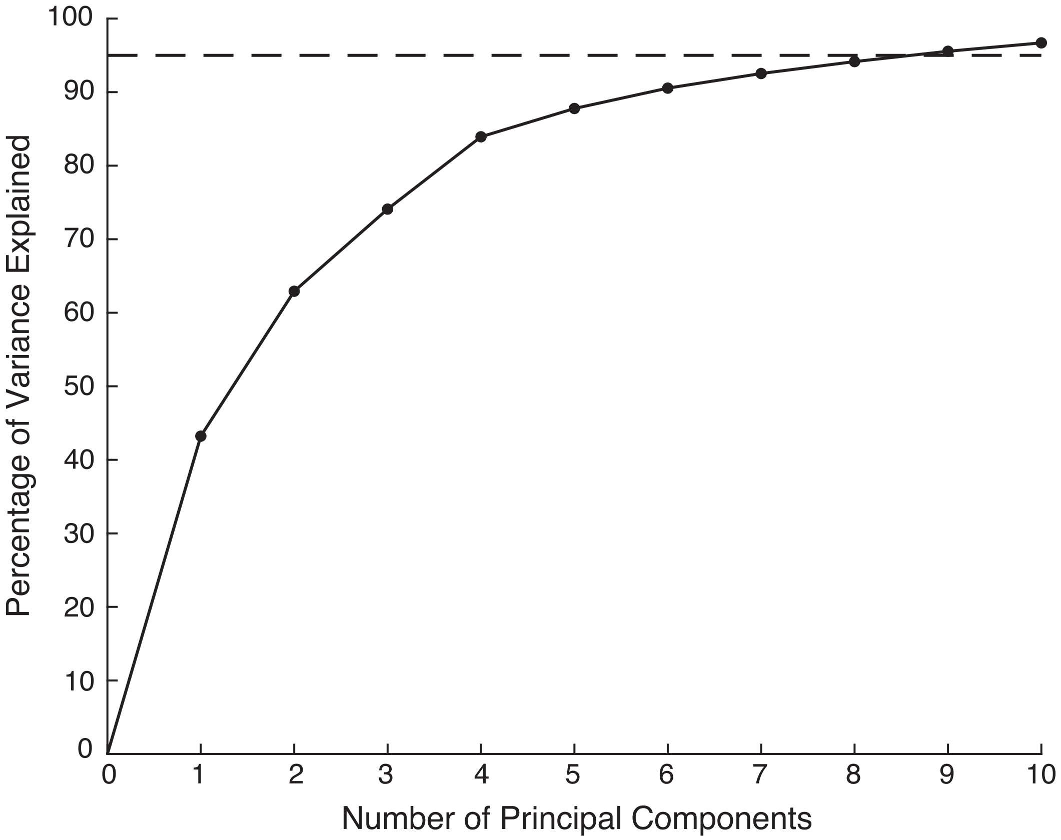

Supplement: S6 Fig — The percentage of variance explained for the first ten components of the principal component analysis performed on the extent of damage to each white matter tract. Dashed line indicates 95% of variance explained. As can be seen, nine principal components explained 95% of the total variance (dashed horizontal line). (PDF) [file pone.0310312.s006.pdf]
