# Supplementary material for: White matter disconnection impacts proprioception post-stroke
Source: PLoS One. 2024 Sep 12;19(9):e0310312. doi: 10.1371/journal.pone.0310312 (PMC11392420; doi:10.1371/journal.pone.0310312)

S7 Fig. Disconnectome Analysis (Analysis uncontrolled for grey matter lesion volume). Voxels with a greater probability of disconnection in participants with Arm Position Matching (APM) Task impairments compared to participants without APM Task impairments. A) Axial view. Images are presented in neurological convention. Numbers above (top) and below (bottom) each slice indicate the axial MNI coordinate. B) Sagittal view. Right (top) and left (bottom) hemisphere are shown. Numbers above (top) and below (bottom) each slice indicate the sagittal MNI coordinate. Note: analysis excludes grey matter lesion volume as a covariate.

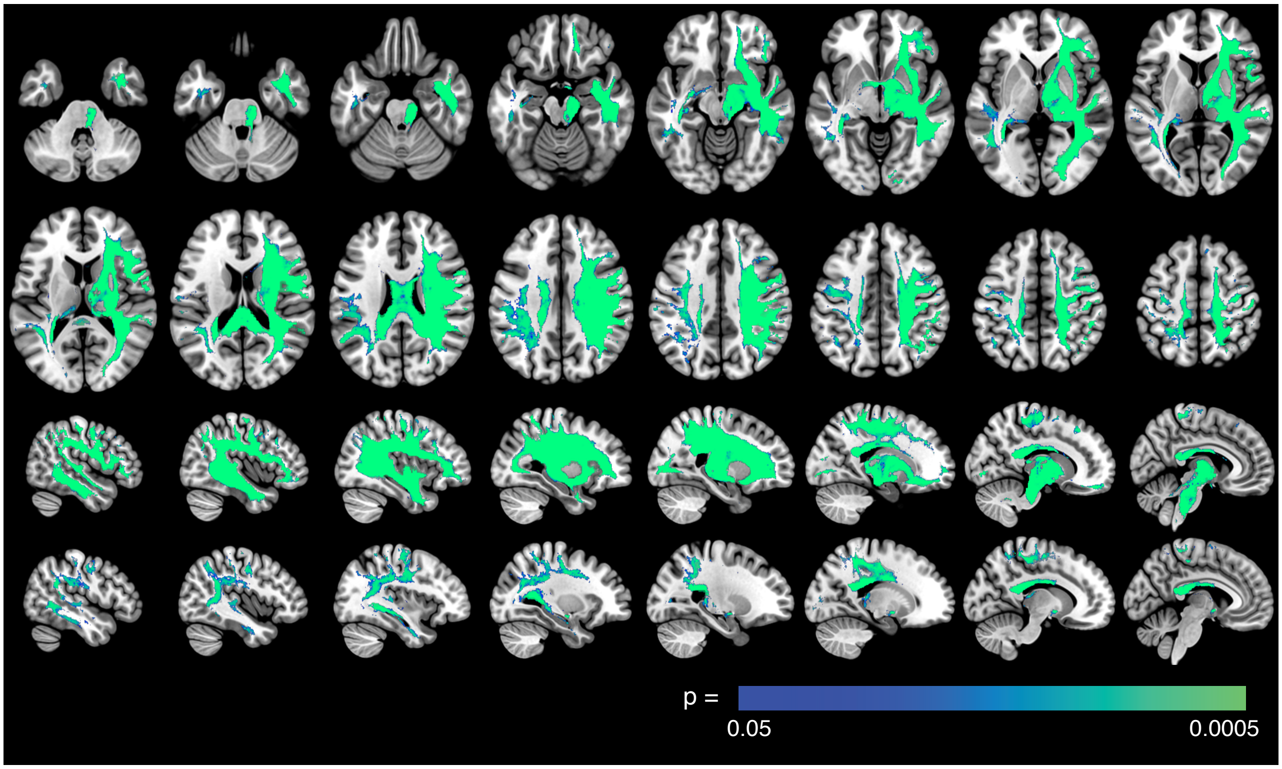

Supplement: S7 Fig — Voxels with a greater probability of disconnection in participants with Arm Position Matching (APM) Task impairments compared to participants without APM Task impairments. A) Axial view. Images are presented in neurological convention. Numbers above (top) and below (bottom) each slice indicate the axial MNI coordinate. B) Sagittal view. Right (top) and left (bottom) hemisphere are shown. Numbers above (top) and below (bottom) each slice indicate the sagittal MNI coordinate. Note: analysis excludes grey matter lesion volume as a covariate. (PDF) [file pone.0310312.s007.pdf]
