# Supplementary material for: White matter disconnection impacts proprioception post-stroke
Source: PLoS One. 2024 Sep 12;19(9):e0310312. doi: 10.1371/journal.pone.0310312 (PMC11392420; doi:10.1371/journal.pone.0310312)
Supplement: S1 Table — The amount of variance explained (%), eigenvalues and principal component loading factors for the lesion load to each white matter tract, on the nine principal components which explained 95% of the total variance. Principal components are ordered according to the amount of variance explained. Variables are ordered according to their weighting on principal component 1. PC = principal component, SLF = superior longitudinal fasciculus, IFOF = inferior fronto occipital fasciculus, CST = corticospinal tract, ILF = inferior longitudinal fasciculus. (PDF) [file pone.0310312.s008.pdf]

S1 Table. Principal component analysis loadings. The amount of variance explained (%), eigenvalues and principal component loading factors for the lesion load to each white matter tract, on the nine principal components which explained 95% of the total variance. Principal components are ordered according to the amount of variance explained. Variables are ordered according to their weighting on principal component 1. PC = principal component, SLF = superior longitudinal fasciculus, IFOF = inferior fronto occipital fasciculus, CST = corticospinal tract, ILF = inferior longitudinal fasciculus

	**PC1**	**PC2**	**PC3**	**PC4**	**PC5**	**PC6**	**PC7**	**PC8**	**PC9**
**Variance Explained**	***43.2***	***19.7***	***11.2***	***9.8***	***3.8***	***2.8***	***2.0***	***1.6***	***1.4***
**Eigenvalue**	***10.4***	***4.7***	***2.7***	***2.4***	***0.9***	***0.7***	***0.5***	***0.4***	***0.3***
**Corpus Callosum**	0.29	0.02	-0.15	0.15	-0.05	-0.05	-0.01	-0.04	0.05
**Grey Matter Lesion Volume**	0.28	-0.13	-0.09	0.09	-0.04	-0.16	-0.17	-0.11	-0.04
**SLF III**	0.25	-0.18	-0.17	-0.18	-0.06	-0.09	0.03	-0.13	0.09
**Arcuate Fasciculus Long Segment**	0.24	-0.21	-0.01	0.05	0.13	-0.16	0.40	0.08	0.00
**Fronto Insular Tract 5**	0.23	-0.15	0.03	-0.27	0.01	0.11	0.02	0.47	0.20
**SLF II**	0.23	-0.08	-0.30	0.07	0.14	0.21	0.12	-0.26	-0.07
**Arcuate Fasciculus Anterior Segment**	0.23	-0.19	-0.24	-0.18	-0.01	0.01	-0.02	-0.04	0.11
**Frontal Aslant**	0.23	0.14	-0.01	-0.16	-0.41	-0.31	-0.09	0.10	-0.28
**Fronto Insular Tract 4**	0.23	-0.10	0.08	-0.33	-0.23	-0.06	-0.14	0.40	0.00
**Fronto Striatal**	0.22	0.19	0.25	-0.20	-0.08	0.07	0.05	-0.31	0.04
**IFOF**	0.22	-0.15	0.22	0.17	-0.35	0.11	-0.14	-0.25	-0.02
**Anterior Thalamic Projections**	0.21	0.27	0.19	-0.10	-0.09	-0.02	0.01	-0.34	0.14
**CST**	0.21	0.24	0.18	-0.10	0.18	0.29	0.34	0.19	-0.07
**Pons**	0.20	0.26	0.22	-0.10	0.10	0.20	0.29	-0.01	0.02
**Arcuate Fasciculus Posterior Segment**	0.19	-0.24	-0.12	0.19	0.21	-0.36	0.33	-0.03	0.00
**Anterior Commissure**	0.18	-0.06	0.38	-0.04	0.37	-0.15	-0.21	-0.19	-0.20
**Frontal Commissure**	0.17	0.35	-0.12	0.10	-0.03	-0.12	-0.12	0.05	-0.05
**Fornix**	0.16	-0.08	0.32	0.14	0.48	-0.11	-0.38	0.21	0.08
**Hand Inferior U Tract**	0.15	-0.10	-0.34	-0.20	0.23	0.49	-0.38	-0.12	-0.05
**ILF**	0.15	-0.20	0.17	0.41	-0.15	0.08	-0.07	0.10	-0.30
**SLF I**	0.14	0.30	-0.22	0.26	0.08	0.03	0.12	0.13	-0.02
**Frontal Superior Longitudinal U Tract**	0.13	0.33	-0.23	0.13	0.07	0.04	-0.13	0.20	-0.48
**Cingulum**	0.12	0.31	-0.12	0.24	0.01	-0.17	-0.22	0.06	0.64
**Optic Radiations**	0.12	-0.15	0.16	0.42	-0.26	0.44	0.05	0.15	0.20
