# Supplementary material for: White matter disconnection impacts proprioception post-stroke
Source: PLoS One. 2024 Sep 12;19(9):e0310312. doi: 10.1371/journal.pone.0310312 (PMC11392420; doi:10.1371/journal.pone.0310312)
Supplement: S2 Table — Principal Component Regression analysis results obtained from the regression analysis between principal component scores for the first 9 principal components and Arm Position Matching Task Scores. The first 9 principal components explained 95% of the variance in white matter lesion load across the sample. Bolded p-values indicate those with significant relationships which survived FDR (5%) correction. Note: Analysis excludes grey matter lesion volume. (PDF) [file pone.0310312.s009.pdf]

S2 Table. Principal Component Regression Results (Analysis uncontrolled for grey matter lesion volume). Principal Component Regression analysis results obtained from the regression analysis between principal component scores for the first 9 principal components and Arm Position Matching Task Scores. The first 9 principal components explained 95% of the variance in white matter lesion load across the sample. Bolded p-values indicate those with significant relationships which survived FDR (5%) correction. Note: Analysis excludes grey matter lesion volume.

	**Estimate**	**SE**	**t-stat**	**p-value**
**Intercept**	6.087 x10^{-16}	0.058	1.041 x10^{-14}	1
**PC1**	0.153	0.019	8.061	**7.772 x10^{-14}**
**PC2**	-0.112	0.027	-4.125	**5.516 x10^{-5}**
**PC3**	-0.046	0.036	-1.291	0.198
**PC4**	0.005	0.038	0.121	0.904
**PC5**	0.179	0.061	2.937	**0.004**
**PC6**	-0.022	0.073	-0.305	0.761
**PC7**	-0.195	0.086	-2.264	0.025
**PC8**	0.034	0.095	0.359	0.720
**PC9**	0.083	0.101	0.818	0.414
