# Supplementary material for: White matter disconnection impacts proprioception post-stroke
Source: PLoS One. 2024 Sep 12;19(9):e0310312. doi: 10.1371/journal.pone.0310312 (PMC11392420; doi:10.1371/journal.pone.0310312)
Supplement: S3 Table — The amount of variance explained (%), eigenvalues and principal component loading factors for the lesion load to each white matter tract, on the three principal components which displayed significant relationships with APM Task Score. Principal components are ordered according to the amount of variance explained. Variables are ordered according to their weighting on principal component 1. PC = principal component, SLF = superior longitudinal fasciculus, IFOF = inferior fronto occipital fasciculus, CST = corticospinal tract, ILF = inferior longitudinal fasciculus. Note: Analysis excluded grey matter lesion volume. (PDF) [file pone.0310312.s010.pdf]

S3 Table. Principal Component Analysis loadings (Analysis uncontrolled for grey matter lesion volume). The amount of variance explained (%), eigenvalues and principal component loading factors for the lesion load to each white matter tract, on the three principal components which displayed significant relationships with APM Task Score. Principal components are ordered according to the amount of variance explained. Variables are ordered according to their weighting on principal component 1. PC = principal component, SLF = superior longitudinal fasciculus, IFOF = inferior fronto occipital fasciculus, CST = corticospinal tract, ILF = inferior longitudinal fasciculus. Note: Analysis excluded grey matter lesion volume

	**PC1**	**PC2**	**PC5**
**Variance Explained (%)**	***41.7***	***20.2***	***4.0***
**Eigenvalue**	***9.6***	***4.6***	***0.9***
**Corpus Callosum**	0.30	0.00	-0.05
**SLF III**	0.25	-0.19	-0.07
**Arcuate Fasciculus Long Segment**	0.25	-0.23	0.13
**Fronto Insular Tract 5**	0.24	-0.17	0.00
**Frontal Aslant**	0.24	0.12	-0.40
**Fronto Striatal**	0.24	0.16	-0.08
**SLF II**	0.24	-0.10	0.13
**Fronto Insular Tract 4**	0.23	-0.13	-0.23
**Arcuate Fasciculus Anterior Segment**	0.23	-0.20	-0.01
**CST**	0.23	0.22	0.17
**Anterior Thalamic Projections**	0.23	0.25	-0.09
**Pons**	0.23	0.24	0.10
**IFOF**	0.22	-0.17	-0.35
**Arcuate Fasciculus Posterior Segment**	0.19	-0.25	0.21
**Frontal Commissure**	0.19	0.35	-0.03
**Anterior Commissure**	0.18	-0.08	0.37
**Fornix**	0.16	-0.09	0.48
**Hand Inferior U Tract**	0.15	-0.11	0.22
**SLF I**	0.15	0.29	0.08
**ILF**	0.15	-0.21	-0.15
**Frontal Superior Longitudinal**	0.14	0.33	0.06
**Cingulum**	0.14	0.31	0.02
**Optic Radiations**	0.12	-0.16	-0.27
